# Sleep Problems in Chronic Inflammatory Diseases: Prevalence, Treatment, and New Perspectives: A Narrative Review

**DOI:** 10.3390/jcm11010067

**Published:** 2021-12-23

**Authors:** Marta Ditmer, Agata Gabryelska, Szymon Turkiewicz, Piotr Białasiewicz, Ewa Małecka-Wojciesko, Marcin Sochal

**Affiliations:** 1 Department of Sleep Medicine and Metabolic Disorders, Medical University of Lodz, 92-215 Lodz, Poland; marta.insmk@gmail.com (M.D.); agata.gabryelska@gmail.com (A.G.); turkiewiczszymon99@gmail.com (S.T.); piotr.bialasiewicz@umed.lodz.pl (P.B.); 2 Department of Digestive Tract Diseases, Medical University of Lodz, 92-215 Lodz, Poland; ewuncia@poczta.onet.pl

**Keywords:** sleep, immunity, psychoneuroimmunology, insomnia, obstructive sleep apnea, insomnia

## Abstract

Epidemiological studies have shown that individuals with sleep problems are at a greater risk of developing immune and chronic inflammatory diseases. As sleep disorders and low sleep quality in the general population are frequent ailments, it seems important to recognize them as serious public health problems. The exact relation between immunity and sleep remains elusive; however, it might be suspected that it is shaped by others stress and alterations of the circadian rhythm (commonly caused by for example shift work). As studies show, drugs used in the therapy of chronic inflammatory diseases, such as steroids or monoclonal antibodies, also influence sleep in more complex ways than those resulting from attenuation of the disease symptoms. Interestingly, the relation between sleep and immunity appears to be bidirectional; that is, sleep may influence the course of immune diseases, such as inflammatory bowel disease. Thus, proper diagnosis and treatment of sleep disorders are vital to the patient’s immune status and, in effect, health. This review examines the epidemiology of sleep disorders and immune diseases, the associations between them, and their current treatment and novel perspectives in therapy.

## 1. Introduction

“Sleep disorder” is a term that encompasses a wide range of chronic disturbances involving sleep and the sleep–wake schedule. There are several classifications in use; International Classification of Diseases, 11th edition mentions a group of disorders or syndromes, such as hypersomnias, insomnia disorder, parasomnias, obstructive sleep apnea (OSA), etc. [1].

Many socioeconomic determinants, such as income, employment, education, and marital status, are suspected to influence the risk for sleep disorders [2]. Shift work, which is common in highly developed countries, is also associated with sleep disorders [3].

Changes to a sleep schedule that are enforced by social and work requirements may increase the prevalence of sleep disorders, which can elevate the risk of developing diseases, such as fibromyalgia, chronic fatigue syndrome, systemic lupus erythematosus (SLE), inflammatory bowel disease (IBD), or rheumatoid arthritis (RA) [4,5].

The aim of this narrative review is to summarize the knowledge on the subject of possible associations between sleep disorders and immune disorders, as well as examine the pathophysiological basis of this relationship.

## 2. Sleep Disturbances in Immune Diseases

Sleep disturbances tend to be more prevalent in patients with immune diseases, as compared to the healthy population, as shown in Table 1. Relation between sleep and immunity appears to be bidirectional: disrupted sleep can result in decreased quality of life and fatigue; it might also exacerbate symptoms such as pain and discomfort, which, in turn, further decreases sleep quality, as discussed below [6,7,8].

We concisely introduce each condition mentioned in this paper to give a better comprehension of the subject. In RA and SLE, mentioned in the introductory part, typical symptoms include joint swelling and pain; in SLE, butterfly rash, as well as hair loss, might also be present. IBD manifestations are primarily gastrointestinal (i.e., constipation alternated with diarrhea, abdominal pain, and rectal bleeding); however, arthropathies might also emerge in the course of the disease. Fibromyalgia, on the other hand, is not an immune disease; it is characterized by dull widespread pain and tiredness. Multiple sclerosis (MS) is an inflammatory demyelinating disease that is characterized by symptoms such as stiffness, tiredness, tingling, and muscle spasms.

What is interesting is that determinants of sleep quality might differ based on the underlying disease and sex. As studies show, the Pittsburgh Sleep Quality Index (PSQI) score of patients with IBD and females with SLE was determined by depressive mood, rather than disease symptoms (sex was not regarded as a factor in the study on IBD) [9,10]. On the contrary, in RA primary factors affecting sleep quality were disease activity and associated pain (90% of the study group was comprised of females) [11]. In MS, poor sleep in males was determined by pain, whereas, in females, by depression and anxiety [12]. Thus, it can be suspected that there is a gender effect on sleep quality in patients with immune-mediated diseases. Gender seems to play an important role in the pathophysiology of sleep disorders in general, affecting prevalence, illness phenotype, etc. [13].

Since the etiology of sleep disturbances is complex, some sleep disorders do not appear to be more prevalent in people with immune diseases or there is vast heterogeneity in studies’ results, as shown in Table 1. Such discrepancies might be a result of treatment (for example, steroids are known to worsen sleep quality), psychiatric disorders (for example, depression, which is known to cause insomnia disorder, is more prevalent in individuals with autoimmune diseases), comorbidities, or socioeconomic factors [2,5,14].

**Table 1:** Prevalence of sleep disturbances in people with immune-mediated diseases and the general population.

	IBD	SLE	RA	Psoriasis	Fibromyalgia	MS	Healthy
Insomnia	22.3–49.6% [9,15]	33.3–71.1% [16,17,18,19]	25.6–70.87% [20,21,22]	5.9–60% [23,24,25]	30.5% [26]	12.5–58% [27,28,29]	10.5–22.6% [18,21,22,23,25]
RLS	7.34–27.8% [30,31,32]	20–40.47% [16,33,34,35]	25–40.35% [36,37]	15.1–40% [23,38,39,40]	33–64% [41,42,43,44]	12.1–57.5% [45]	4.8–14.2% [23,30,31,33,34,35,39,40]
OSA	13% [5]	23–42% [34,46,47]	26.3–80% [48,49]	36–81.8% [23,50,51]	24.4–52.6% [52]	21–78% [53,54,55]	1.5–38% [23,34,50,51,56]
High PSQI	43.6–67.5% [9,30,57]	55.4–76.7% [10,58,59,60,61,62,63]	38.5–86.5% [64,65,66,67,68,69]	78.3–90% [40,70]	96% [71]	44–65% [12,72,73]	13.4–33.9% [9,30,40,66,70,74]

Abbreviations: inflammatory bowel disease (IBD), obstructive sleep apnea (OSA), Pittsburgh Sleep Quality Index (PSQI), rheumatoid arthritis (RA), restless leg syndrome (RLS), systemic lupus erythematosus (SLE). PSQI cutoff point for high PSQI was >5 or >6, depending on the study. Cutoff point for OSA was Apnea/Hypopnea Index ≥5 or >5. Prevalences are based on the references provided for each number.

Sleep disorders are not specific to a given disease. However, different symptoms of the underlying disease result in a different profile of sleep disorders in certain groups of patients.

Joint degeneration, the primary manifestation of RA, might contribute to the particularly high risk of OSA in this group—the hazard ratio (HR) for OSA, as compared to the general population, is reported to be as high as 2.00 and 1.51 for males and females respectively, with prevalence as high as 80% [49]. This might be explained by pathologies of the temporomandibular and cricoarytenoid joints, as well as cervical spine instability, which can obstruct the airflow, leading to periods of apnea and sleep fragmentation [75].

Demyelination of neurons, causing damages to the cervical cord in MS, might contribute to the high prevalence of restless leg syndrome (RLS) in this group. It was shown that patients with greater cervical spine damage have an increased RLS prevalence [76]. The mechanism behind this association is not fully clear; however, it might be related to the damage of the descending cerebrospinal inhibitory pathway [77]. Impairment of this tract results in greater excitability, causing RLS [77].

As shown in Table 1, data on the prevalence of OSA in IBD patients are scarce; however, it seems to be similar to that of the general population, as well as lower in comparison with other immune-mediated diseases. A possible explanation of this finding is that obesity, a strong risk factor for OSA, tends to be less prevalent in IBD (18%) as compared to the general population (28%) and other diseases (32–50% in fibromyalgia, 20–31.6% in RA, 28.3% in MS) [5,78,79,80]. To the best of our knowledge, there has been no research on the differences in OSA prevalence between ulcerative colitis (UC) and Crohn’s disease (CD) patients. This subject requires further studies.

Psoriasis is a chronic skin disease that causes abnormal cell proliferation and the formation of lesions. Extracutaneous manifestations, such as psoriatic arthritis, might also occur. The risk for OSA in psoriasis appears to be associated with disease severity, as well as the presence of psoriatic arthritis, with incidence rate ratios rising for mild, severe, and rheumatological forms. One factor contributing to this finding might be that the degeneration of joints (e.g., cricoarytenoid and temporomandibular) can obstruct the airflow in patients with this form of psoriasis. Moreover, as the study shows, psoriasis increases the risk of obesity within 10 years, which further exacerbates the risk of apneas/hypopneas [50].

Sjögren’s syndrome (SS) is a disease affecting salivary and lacrimal glands, causing impairment of fluid secretion. SS symptoms include dry mouth, eyes, pain, and fatigue. Behcet’s disease is a condition that might manifest in tissues and blood vessels, causing canker sores, genital ulcers, uveitis, etc. Inflammatory reactions, which are present in both diseases, can spread to the upper airways, rendering them prone to collapse and airflow obstruction [81]. This process might account for the finding that both SS and BD patients have an increased risk for OSA, with males’ risk being more than two times as high as females’ [81].

### 2.1. Sleep Assessment Methods

Certain sleep disorders, such as insomnia, are diagnosed by using subjective methods (questionnaires and sleep diary), while others require an objective approach (actigraphy, polysomnography (PSG), or multiple sleep latency test).

PSG is considered to be the most reliable method in assessing sleep time and fragmentation. It monitors general somatic parameters, such as cardiac rhythm, breathing (oro-nasal flow, respiratory effort), oxygen saturation, and sleep architecture.

Sleep architecture is composed of cycles between non-rapid eye movement (NREM) and rapid eye movement (REM) sleep. NREM has four stages; stages III and IV are often collectively described as slow-wave or deep sleep, due to distinctive pattern on the electroencephalographic record. Other parameters used to describe sleep are total sleep time (time spent asleep), sleep efficiency (ratio of time spent asleep to time spent in bed), sleep latency (time between sleep-initiating behavior, e.g., lights out to the first sleep stage), and wake after sleep onset (time spent in a wakeful state after sleep onset), reflecting sleep fragmentation.

Actigraphy, another objective method, measures sleep parameters (such as total sleep time, sleep efficiency, sleep latency, wake after sleep onset, and number of awakenings) indirectly through analysis of a movement record (patient wears an actigraph, usually on the wrist, for an extended period of time in his/her natural environment). Actigraphy might be utilized in the diagnosis of circadian rhythm disorders, providing a dependable record of sleep patterns.

Questionnaires are one of the most common subjective sleep assessment methods. They tend to have high sensitivity for sleep disorders (73.0–97.7%) [58]. Questionnaires are widely used in the assessment of overall sleep quality (for example, the Pittsburgh Sleep Quality Index (PSQI)) and evaluation of specific symptoms or disorders (Epworth Sleepiness Scale for excessive daytime sleepiness (ESS), Athens Insomnia Scale (AIS) or Insomnia Severity Index for insomnia, Ullanlinna Narcolepsy Scale for narcolepsy, etc.) [82,83,84,85,86]. Most studies on the prevalence of sleep disruptions focus on the general sleep quality, as specific disorders require a complex long-term diagnostic process.

A sleep diary is a subjective method characterized by constant data collection, providing a record enabling clinicians to track night-to-night changes in sleep parameters and determine progress in therapy, trends, and the association of sleep quality with other factors.

Subjectively measured sleep parameters might be skewed by many factors, such as sleep state misperception (perception of sleep as wakefulness), which was found to be common in patients with chronic insomnia (26.4% in patients with primary insomnia, diagnosed according to the Diagnostic and Statistical Manual of Mental Disorders IV) [87]. It is necessary to emphasize that the sleep state misperception (or paradoxical insomnia) remains controversial, with some studies showing that viewing it as a clinical entity has no empirical support [88,89].

Questionnaires’ scores might not reflect objective sleep parameters. As one study shows, global sleep assessment using PSQI did not correlate with objective sleep duration, efficiency, or latency measured by actigraphy [90]. A similar study also reported a major difference between sleep time assessed by using actigraphy and estimated by the participant [91]. In general, it can be stated that subjective sleep quality has a rather tenuous correlation with objective parameters [92].

Sleep diaries, in contrast to questionnaires, are less prone to be biased by flawed recall: patients are meant to log sleep data daily; however, nocturnal awakenings might not be remembered. Some researchers report that sleep diaries might overestimate total sleep time, as well as sleep onset latency [93,94].

It appears that the use of sleep diaries in future studies on the relationship between immune diseases and sleep disorders might allow the researchers to collect more data, unrestricted to a given type of sleep disruption, thus enabling more versatile analysis. Both questionnaires and sleep diaries are subjective methods of sleep evaluation; thus, data obtained are skewed by the patient’s perception, as was discussed above. However, they can reliably reflect the decreased quality of sleep, which itself requires an intervention.

### 2.2. Insomnia

Insomnia is a disorder characterized by problems in initiation, maintenance, and too early awakening, resulting in subjectively impaired daily functioning. Standard tools for diagnosing insomnia are interviews and questionnaires, which assess the duration and frequency of symptoms.

As shown in Table 1, it is the most prevalent sleep disorder in patients with immune-mediated diseases, such as SLE and RA. Notably, in diseases such as IBD or psoriasis, the data on insomnia-disorder prevalence are ambiguous; some studies indicate a lower prevalence in this group, as compared to the general population.

Insomnia has many contributors, such as psychiatric disorders, bladder dysfunctions, other disease symptoms, and medications (for example, anticonvulsants, beta-agonists, psychostimulants, amphetamines, steroids, and dopamine agonists) [95,96,97,98,99]. Studies on subjects with psoriasis suggest that insomnia disorder is associated with pruritus and pain [23]. However, in one study on IBD patients, there was no difference in AIS (scale used specifically for insomnia diagnosis) score between patients in exacerbation and remission, suggesting that disease symptoms had little impact on insomnia symptoms [9]. In SLE, insomnia symptoms were more prevalent in patients with renal involvement, which might indicate a bidirectional relation between disease severity and this sleep disorder [17]. Moreover, in this study group, individuals with insomnia tended to have a higher level of perceived stress, which has been associated with disease exacerbation [17].

Sleep loss might exert an influence on the course of the disease, as well as the overall patient’s condition. In one study performed on RA patients, even partial sleep deprivation (SD) was shown to exacerbate the severity of pain, number of painful joints, and fatigue [74]. According to Ananthakrishnan et al., sleep disruptions are a risk factor for disease exacerbation in CD (odds ratio (OR), 2.00; 95% confidence interval (CI), 1.45–2.76) [97].

### 2.3. Sleep-Disordered Breathing

“Sleep-disordered breathing” is a term that encompasses central sleep apnea, obstructive sleep apnea (OSA), and other mixed apneas.

Central sleep apnea is characterized by pauses in breathing due to the inability to control respiration of central origin. It can manifest as Cheyne–Stokes breathing, idiopathic central sleep apnea, narcotic induced central sleep apnea, or be a result of PHOX2B mutation, as is the case in central congenital hypoventilation syndrome [100]. Sleep apneas are diagnosed during PSG, through assessment of the apnea/hypopnea index. The primary difference between central and obstructive sleep apnea is the respiratory effort, which, in the case of central sleep apnea, is not observed during apneic episodes.

Episodes of apnea/hypopnea might occur during REM, NREM, or both. Decreased muscle tone during REM is most likely responsible for airway obstruction during REM [101]. Breathing in NREM, on the other hand, is controlled primarily by chemoreception [101]. As one study shows, ventilatory instability caused by reduction of ventilation during the transition from sleep to wakefulness (accompanied by an appropriate change in the PaCO2 level) might contribute to the emergence of the NREM-predominant phenotype of sleep disordered breathing [101].

OSA is a disorder affecting 9–37% men and 4–50% women, with men being consistently more affected than women [95,102]. It is characterized by apneic episodes during sleep, resulting from restricted airflow through the upper airways [96]. There are three types of obstructive breathing events: apnea, hypopnea, and respiratory effort-related arousal. Risk factors for OSA include obesity, male sex, increased neck circumference, middle age, enlarged tongue, enlarged tonsils, retro/micrognathia [96]. Consequences of this disorder are sleep fragmentation, increased daytime sleepiness, impaired concentration, fatigue, etc. [96].

It appears that sleep-disordered breathing renders people more susceptible to immune-mediated disorders. OSA patients are at greater risk of diseases, such as psoriasis (prevalence 8.7%, compared to 2% in the general population), RA (adjusted hazard ratio (aHR) 1.33), SS-aHR 3.45), and BD-aHR 5.33 [81,103,104]. The interaction between the two, as in many such cases, might be bidirectional—the influence of immune-mediated diseases on the OSA development has already been discussed above. Another aspect of this relation is the influence of OSA on the immune system, which remains, to some extent elusive. Intermittent hypoxia, which might increase hypoxia-inducible factor 1 (HIF-1), is proposed to influence the T helper (Th) 17 cells/T regulatory (Treg) cells balance, an important factor in the development of immune-mediated diseases, such as psoriasis, RA, IBD, and MS [105,106]. HIF-1 might inhibit Treg production, as well as promote the formation of pro-inflammatory Tregs, which produce interferon ɣ (INF ɣ) [105]. HIF-1 also stimulates the production of Th17 (and subsequently interleukin (IL) 17), shifting the balance in favor of immune response instead of tolerance [105].

It has also been suggested that hypoxia causes the deposition of uric acid in cells, which has been linked to cell-mediated autoimmune reactions [107]. Individuals with OSA were also shown to have elevated levels of pro-inflammatory cytokines, indicating dysregulation of the immune system [107].

According to one study, treatment with continuous positive airway pressure (CPAP) decreases HR to 0.22 for RA and 0.51 for other autoimmune diseases, which were not specified by the authors [103].

### 2.4. Circadian Rhythm

Circadian rhythm is a 24-h endogenous rhythm controlled by the central master clock—the suprachiasmatic nucleus, as well as peripheral clocks in tissues [108]. Circadian clock genes show oscillatory expression patterns, forming feedback loops, which enable autoregulation [108]. They include CLOCK, brain and muscle ARNT-Like (BMAL1), RAR-related orphan receptor alpha (RORa), period circadian regulator (PER) 1 and 2, and cryptochrome (CRY) [108].

It is well evidenced that there is a complex and bidirectional relation between inflammation and clock genes. According to one study performed on mice, RORa was necessary to decrease the severity of inflammation through modulation of transcription of nuclear factor kappa-light-chain-enhancer of activated B cells (NF-κB) [109]. It has been shown that BMAL1-deficient mice had arrhythmic and decreased cell proliferation in the intestines, resulting in impaired tissue regeneration [110]. Expression of PER 1 and 2 is inhibited by the tumor necrosis factor (TNF), while BMAL1 is induced by this cytokine in synovial cells, contributing to cell proliferation and subsequent progression of RA [111,112]. PER2 and CRY 2 expressions are decreased by IL-6 and increased by TNF in human monocytic cells [113]. In spontaneously immortalized monocyte-like cells, levels of PER2 and CRY2 were significantly increased after the anti-TNF treatment [113]. TNF exerts this regulatory effect on gene expression perhaps through modulation of the Ca2+ influx, as well as through interaction with the D-box (regulator of transcription) in the case of PER2 [112,114,115]. It might be suspected that increased levels of some pro-inflammatory cytokines, resulting from disrupted sleep, further enhance changes of clock genes’ expressions caused by the disease, creating a vicious circle.

Regarding epidemiological studies, Sonnenberg et al. suggested that alteration of the circadian rhythm, among others caused by artificial light or shift work, might be associated with increased risk for IBD [116]. Indeed, patients with IBD had decreased expression of clock genes in white blood cells and intestinal mucosa [117]. Immune-mediated thyroid diseases have also been linked to shift work [118]. As another study shows, advanced/delayed sleep phase syndrome, irregular sleep–wake patterns are more common in patients with MS [119]. Moreover, drug chronotherapy (a form of therapy in which the administration of drugs is based on the 24 h circadian rhythm) with glucocorticosteroids and disease-modifying anti-rheumatic drugs was shown to be beneficial in RA [120,121,122]. This approach also appears promising in other diseases, such as SLE, but more studies would be desirable [123].

### 2.5. Restless Leg Syndrome

RLS is described by the patients as an unpleasant, crawling feeling in the legs [36]. Diagnosis is usually reached based on reported symptoms, using scales such as RLS-6 or The International Restless Legs Syndrome Rating Scale, although RLS can also be detected during PSG. It affects around 5–15% of the population and women twice as often as men [36]. It is a common condition among patients with immune-mediated and chronic inflammatory disorders, such as RA (30% of patients have RLS), MS (12.12–57.50%), CD (17.6%), and UC (21.7%) [36,124,125,126]. In the case of ankylosing spondylitis (AS), a form of inflammatory arthritis that primarily affects the spine and can produce symptoms similar to RLS (tinging in the lower limbs), reported prevalence is as high as 30.8% [126]. Assessing the prevalence of RLS in MS patients is particularly difficult because both conditions show similar symptoms (crawling sensation, etc.). This might explain discrepancies between studies’ results on the prevalence of RLS in MS—many cases might not have been reported. The pathophysiology of this condition is not fully understood. Studies suggest its association with the dopaminergic system and low iron levels in the substantia nigra. [127] Low iron level in the blood, also linked to RLS occurs in many MS and IBD patients [76,128].

### 2.6. Summary

There is a complex and bidirectional relation between sleep disorders and immune-mediated diseases. As studies show, many of them increase the risk of developing sleep disorders and vice versa. One aspect of this relation is that the influence of the disease on sleep disorders depends on the pathophysiology, symptoms, and sequelae of a given condition (e.g., MS causes crawling feeling in the legs that are similar to RLS; RA causes joint pathologies, which might increase the risk of OSA; etc.). Another aspect, changes in the immune system caused by sleep disorders, is still a subject for research. It seems that sleep disorders might negatively affect both the risk of disease development (e.g., OSA increases the aHR for BD, SS, and psoriasis) and course (for example, causing more frequent relapses).

## 3. Sleep and Inflammation

Sleep is vital for developing proper immune and inflammatory responses. As one study shows, it might facilitate the homing of the Th cells to lymph nodes, where they can come into contact with an antigen, differentiate, and produce pro-inflammatory cytokines [129]. Innate immunity, involving the production of cytokines such as IL-6 and IL-1β, is affected by sleep, as well: it facilitates an increase in the production of reactive oxygen species, a number of circulating monocytes, and monocyte trafficking [130]. It is important to see the relation between sleep and immune response as bidirectional.

The influence of cytokines on sleep is often complex and difficult to determine, as studies’ results are often heterogeneous. This subject is still being studied and decisive conclusions, as of now, cannot be drawn. In our review, we discuss the influence of TNF, IL-6, IL-1β on sleep, as all of them have a major role in the inflammatory response.

IL-6 is a pro-inflammatory, pyrogenic cytokine [131]. Some authors report increased levels of IL-6 in both short and extremely long sleep, as well as immune diseases such as RA, CD, or psoriasis [131,132,133]. According to Rohleder et al., when administered to humans, it was shown to decrease slow-wave sleep (SWS) during the first half of the night and increase in the second half [131]. This might be to some extent the effect of short-term stimulation of cortisol production (physiological peak of which occurs around the sleep offset) by this cytokine [131]. However, increased IL-6 in general correlates with decreased SWS [134]. Vgontzas et al. have reported that SD during the early period of the night affects the pattern of secretion of the cytokine: the peak occurs later, and serum cytokine levels stay relatively low [135]. IL-6 also promotes the production of Th17 cells, affecting Treg/Th17 equilibrium, enhancing pro-inflammatory response [136]. Accompanied by other cytokines, it might also impair the regulatory function of Tregs, advancing inflammation [136]. IL-6 might contribute to sleep disorders such as RLS. It induces hepcidin production in hepatocytes, which decreases iron absorption in the intestines and inhibits iron release from the macrophage, leading to hypoferremia, which is associated with fatigue, irritability, and RLS [137].

IL-1β is a pyrogenic pro-inflammatory cytokine. Studies have revealed its involvement in the pathophysiology of immune diseases, such as RA and SLE, as well as immune-mediated thyroid diseases [138]. According to Hahn et al., it might enhance non-rapid eye movement (NREM) sleep and sleep intensity [139]. This effect is thought to be mediated by the IL-1 receptor [139]. SD was reported to increase IL-1β serum levels, which could potentially promote processes such as the production of plasmocytes, dendritic cells, and M1 macrophages, as well as the production of Th cells and cytotoxic T lymphocytes [140,141]. An increase in IL-1 β serum levels after SD suggests the involvement of inflammasome. The inflammasome is a structure consisting of caspase-1, an adaptor protein, and the NLR family pyrin domain containing 3 (NLRP3), which cleaves IL-1, forming IL-1β and α.

As one study shows, NLRP3 knock-off (KO) mice showed a different response to SD than wild-type (WT) mice: their NREM intensity (as measured by delta power) did not differ significantly from the baseline, whereas in WT mice this parameter was increased [142]. It is interesting that rapid eye movement sleep (REM), usually not associated with IL-1 β: REM episode duration and frequency was also altered only in WT mice [142]. This suggests that the inflammasome is involved in the shaping of the sleep architecture during inflammation. Inflammasome also partakes in the process of IL-18 formation, which stimulates the production of INF ɣ by T and natural killer cells. INF ɣ is associated with immune-mediated diseases, such as SLE or CD [141].

TNF is a pro-inflammatory cytokine. Its increased levels have been described in sleep disorders such as OSA, as well as immune diseases, for example, RA, CD, and psoriasis [143,144]. It stimulates the production of pro-inflammatory mediators, helping in the recruitment of immune cells, suppresses Tregs, enhances nociception in the central and peripheral nervous system, and might stimulate enzymes that contribute to tissue degeneration [145]. TNF appears to promote NREM, while, in higher doses suppressing REM in animal studies [146]. According to one study on humans, a higher level of TNF after intravenous endotoxin administration was associated with increased sleep propensity [147]. According to another study, after central administration, TNF enhanced sleep intensity in NREM [139]. It appears that serum levels of TNF are not affected by SD or sleep disturbances, although the results of the studies are heterogeneous [132]. Imeri et al. have suggested that such alterations in sleep architecture (REM suppression and NREM increase) might facilitate fever: energy is spared through long NREM, whereas shorter time spent in REM allows for shivering.

Attenuation of inflammation can be mediated by the hypothalamic–pituitary–adrenal axis (HPA), a highly complex set of endocrine interactions between the hypothalamus, the pituitary gland, and the adrenal gland, through cortisol production. Its function can be disrupted by sleep disorders. Insomnia disorder does not seem to impair HPA function; however, in those individuals, a non-physiological increase in the evening level of cortisol was observed [148]. It needs to be mentioned that this subject remains controversial and more studies are warranted to determine the association between the two. Shift work, a factor associated with sleep disorders, can also influence the diurnal secretion of cortisol (HPA activity marker) [3]. One longitudinal study on young (mean age 30) subjects showed that waking levels of cortisol are higher in shift workers, total secretion is increased, and the decrease occurs faster [149]. Other studies show opposite results, with waking cortisol levels being decreased and slope flatter (which might also be a sign of HPA dysfunction) [149,150]. The authors of the study explain those differences as a result of the higher age of participants, as aging might be associated with changes to the diurnal cortisol secretion [149,151]. Patients with RLS do not show abnormalities in the HPA function and cortisol secretion; studies on the subject are unequivocal [148]. HPA dysfunction appears to be associated with OSA and its comorbidities [152]. Kritikou et al. have shown that, in non-obese males and obese females with OSA, cortisol levels are increased compared to controls [153]. According to Minami et al., an increase in adrenal gland size was associated with sleep fragmentation in OSA patients, thus further supporting the association between the HPA axis and OSA [154]. CPAP treatment, a standard therapy for OSA, also appears to lower cortisol levels [152]. The role of the HPA axis in OSA patients requires further studies, as its understanding might help to alleviate comorbidities, such as hypertension.

**Figure 1:**
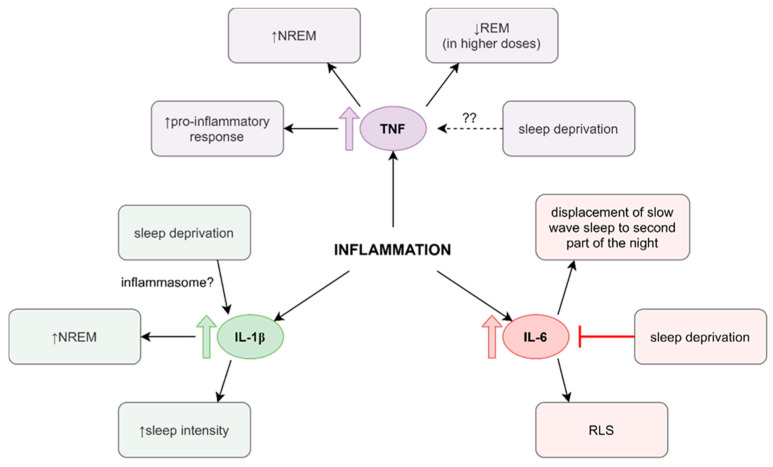
Sleep and inflammation. Notes: Inflammation is associated with relatively high levels of interleukin (IL) 6, IL-1β, and tumor necrosis factor (TNF). IL-6 might promote slow-wave sleep in the second half of the night while suppressing it in the first half. It might also contribute to restless leg syndrome (RLS) through stimulation of hepcidin production, which can cause iron deficiency. Sleep deprivation (SD) early in the night might cause relatively low IL-6 levels and change its pattern of secretion. IL-1β might improve non-rapid eye movement sleep (NREM) length and sleep intensity. Its levels increase after SD, suggesting the influence of SD on inflammasome, a potentially interesting subject for future studies. TNF stimulates immune response through suppression of Treg lymphocytes, recruitment of the immune cells, etc. It appears to promote NREM while suppressing REM. Studies’ results on the influence of SD on TNF levels are unequivocal.

To summarize, inflammation and sleep have a complex and bidirectional relation, some aspects of which were summarized in the Figure 1. Acute, as well as chronic, SD might lead to an increase in pro-inflammatory mediators, disrupting various immune functions, which might be a significant risk factor for immune-mediated diseases.

## 4. Therapy with Monoclonal Antibodies

Therapy with monoclonal antibodies is a form of therapy targeting specific antigens in the treatment of immune-mediated conditions and cancers. It was a major breakthrough in medicine, enabling better management of chronic inflammatory diseases. What is interesting, monoclonal antibodies might be utilized in the therapy of psychiatric diseases, such as depression [155]. There, anti-IL12/23 and anti-IL-6 brought about improvement in low mood and anhedonia, which could not be fully ascribed to the amelioration of symptoms of underlying immune-mediated disease [155].

Monoclonal TNF blockers, e.g., adalimumab and infliximab (IFX), are a relatively safe and effective way of managing diseases such as CD [156,157]. Moreover, they appear to influence sleep and fatigue. In a study on patients with spondylarthritis treatment with another anti-TNF antibody, golimumab was shown to improve sleep quality measured by using Jenkins Sleep Evaluation Questionnaire [158]. Improvement was seen only in patients, whose clinical parameters (i.e., pain, overall functionality, and signs of inflammation) were better compared to the baseline [158]. Thus, this increase in sleep quality might be attributed to pain amelioration [158].

A study on the influence of adalimumab on sleep quality in patients with AS brought similar results: treatment improved sleep adequacy and reduced somnolence (reported subjectively by the patients, the assessment was performed by using Medical Outcomes Study Sleep Scale) [159]. Sleep quality improvement was also associated with improvement in other parameters: back pain, C-reactive protein concentration, etc. [159].

In another study conducted on RA patients treated with IFX, it was observed that IFX administration improved sleep structure by decreasing the number of arousals and sleep latency time of phases I and II NREM and increasing sleep efficiency, duration of REM, and SWS in the night following the drug administration. Psychomotor tests were performed within 15–18 h after IFX infusion [160]. Vigilance was enhanced; however, daytime sleepiness remained the same [160]. There was no improvement in clinical parameters, such as a number of swollen/tender joints and morning stiffness, so the mentioned changes cannot be attributed to disease amelioration [160].

Other therapies with monoclonal antibodies also yield promising results. Subjective sleep quality and daytime sleepiness (measured using PSQI and ESS) improved in RA patients after the therapy with IL-6 antibody tocilizumab (TCZ) [161]. As another study shows, therapy with TCZ resulted in a reduction of fatigue, as measured by Fatigue Scale The Functional Assessment of Chronic Illness Therapy [162]. Disease activity was not significantly associated with daytime sleepiness or sleep quality, thus suggesting that blocking of this cytokine alone can improve sleep.

Therapy with Ixekizumab, an IL-17A antibody, in patients with AS improved fatigue and sleep quality. Those effects were most evident in subjects whose clinical status has improved; thus, the aforementioned changes can be ascribed to disease amelioration [163].

Therapy with monoclonal antibodies might decrease inflammation, thus ameliorating pain, which itself can account for better sleep quality. Since proinflammatory cytokines are able to influence the sleep structure, changes in their levels following the therapy might affect sleep independently of clinical outcomes.

## 5. Treatment of Patients with Chronic Inflammatory Diseases and Comorbid Sleep Disorders

### 5.1. Cognitive–Behavioral Therapy for Insomnia

Cognitive–behavioral therapy for insomnia (CBTi) is the preferred treatment in this sleep disorder, as it yields moderate to large effects, sustained for a long period of time, with no adverse side effects, as is the case in the pharmacological approach. CBTi involves relaxation, sleep restriction, stimulus control, and cognitive techniques, such as paradoxical intention (encouraging the patient to stay awake in bed, to face the anxiety of being unable to fall asleep), management of dysfunctional thoughts [164]. Moreover, CBTi was shown to reduce inflammation. In a study conducted by Irwin et al., individuals who applied this form of treatment had reduced CRP, proinflammatory gene expression, and monocyte production of pro-inflammatory cytokines, as compared to the baseline [165].

### 5.2. Hypnotic Drugs

Hypnotic drugs are used more frequently by patients with the chronic inflammatory disease compared to the general population. According to Sochal et al., about 15% of IBD patients take hypnotic drugs [9]. In a Dutch study, benzodiazepine and opioid use were respectively 2- and 5-fold more frequent in IBD patients compared to the controls [166]. In a study performed on SLE patients, 9% of them used anxiolytics/hypnotics compared to 5% in the control group [167]. In MS, 47% of patients use hypnotic drugs at least occasionally [168]. These data show how widespread the treatment of sleep disorders in individuals with immune-mediated diseases is; education of both physicians and patients on the subject is necessary to minimalize the risk of substance (opioid, benzodiazepine, and Z-drugs) abuse.

Benzodiazepines are commonly prescribed medications for insomnia disorder. Their prolonged (longer than 4 weeks) use is not recommended by the European Sleep Research Society [169]. They are highly addictive, might impair memory, and cause fatigue [170]. Benzodiazepines might be particularly harmful to UC patients: in a rat model of UC, those drugs were shown to dose-dependently increase mucosal damage [171]. Due to the highly addictive potential and the possibility of disease exacerbation, treatment with benzodiazepines should be avoided in patients with IBD. Z-drugs have a similar pharmacological profile to benzodiazepines. Eszopiclone is an example of a Z-drug intended for insomnia treatment. It was shown to improve parameters such as total sleep time, sleep latency, and quality in RA individuals [172]. Unfortunately, it is not intended for long-term use: after two weeks, dependence or tolerance might arise.

Another group of drugs used to treat insomnia disorder is tricyclic antidepressants (TCA), such as doxepin. Doxepin was approved for the treatment of long and short-term insomnia in March 2010 in doses 3 and 6 mg. It is thought to exert its effect through the blocking of the H1 receptor in the tuberomammillary nucleus. Effect size in regard to sleep duration and maintenance ranges from small to medium; however, it does not appear to improve sleep initiation [173]. Side effects of doxepin are negligible, with the two most common being somnolence and headache, making it unlikely to aggravate the symptoms of patients with immune diseases. Moreover, low doses of TCAs were shown to have an ameliorating effect on the residual IBD symptoms [174]. However, a subset of patients with the major depressive disorder might not benefit from this treatment; according to a retrospective case series, they do not appear to change the sleep maintenance and onset [175]. Doxepin is also not approved for insomnia treatment in many European countries, such as Germany, the United Kingdom, and Poland. Mianserin, a tetracyclic antidepressant also proved effective in primary insomnia [176]. Trazodone, a triazolopyridine derivative is an antidepressant commonly used in the treatment of primary and secondary insomnia. It is used in both depressed and otherwise healthy patients.

A novel approach to the treatment of insomnia disorder is orexin receptor antagonists, such as suvorexant and daridorexant. They act by blocking orexin receptors which promote wakefulness. It was shown to improve sleep efficiency, maintenance, and onset [177]. It does not appear to have major side effects. Those qualities render it fit for patients with chronic inflammatory diseases, but more studies on the subject would be desirable. Orexin receptor antagonists are not available in many countries. As of 2021, daridorexant awaits European approval as the first drug in this class on the market, whereas suvorexant has been approved only in the United States, Japan, and Australia.

### 5.3. Melatonin

Melatonin is a neurohormone produced in the pineal gland. It gives feedback to the suprachiasmatic nucleus (central circadian master clock) by binding to the melatonin 1 (MT1) and MT2 receptors, thus contributing to the regulation of the circadian rhythm.

Melatonin might be useful in the treatment of disorders related to the dysregulation of the circadian rhythm. In delayed sleep phase syndrome, it was shown to improve sleep latency and modify the circadian rhythm [178]. Melatonin is not effective in improving sleep parameters, such as total sleep time; nevertheless, the MT1/2 agonist ramelton was approved for insomnia disorder treatment in the USA and Japan. It appears to improve total sleep time, sleep quality, efficiency, and latency [179]. The clinical significance of those effects is small; however, when considering negligible side effects, it might be a good therapeutic option [179]. Agomelatine, another synthetic MT1/2 agonist and 5-hydroxytryptamine subtype 2C (5-HT2C) weak antagonist, was shown to be useful in improving sleep parameters such as total sleep time and sleep efficiency in patients with the major depressive disorder [180]. Its safety profile is satisfactory, as well [181].

Melatonin might exert either a pro- or anti-inflammatory effect. It acts as a reactive oxygen species neutralizer and apoptosis inhibitor and might block the expression of NF-κB, TNF [182]. In a study on a mice model of the lung, inflammation melatonin was able to lower the level of IL-6 and TNF in animals administered with lipopolysaccharide and subjected to SD [183]. Another study on sleep-deprived mice with IBD yielded similar results [182]. In a study on SLE, mice melatonin was able to counteract the increase in anti-ss-DNA and anti-histone antibodies, as well as ameliorate the disease’s destructive effects on the kidneys [184]. The pro-inflammatory properties of melatonin might be exemplified in RA. Some authors claim that melatonin treatment might have an adverse effect on RA patients due to the promotion of immune responses (both cell-mediated and humoral), as well as stimulation of pro-inflammatory cytokine production [185]. However, in a clinical trial conducted by Forrest et al., 6 months of melatonin administration (10 mg/day) did not affect the level of pro-inflammatory cytokines [185]. Peroxidation markers decreased and erythrocyte sedimentation rate and neopterin (proposed as a marker of inflammation, disease activity score in RA) increased, but clinical symptoms did not change [185]. Sleep was not evaluated in this study. The authors concluded that melatonin is neutral for RA patients [185].

Melatonin appears to be more useful in circadian rhythm disorders rather than insomnia disorder. Administration of both melatonin and MT agonists does not have major adverse effects on health, so this might be a good treatment option for people with chronic inflammatory diseases. According to Chojnacki et al., melatonin might be used as an adjuvant treatment in order to prolong the remission in UC patients [186]. It also appeared to have a favorable impact on the psychological well-being of the study’s participants, although without conspicuous improvements in anxiety and depression scores [186]. Further studies on the subject of the effectiveness of melatonin use in chronic inflammatory diseases are warranted.

### 5.4. Probiotics

Modulation of the microbiota composition through probiotic administration was shown to have a beneficial effect in immune-mediated diseases. In SLE mice administrated with probiotics, IL-10 was increased, IL-6 diminished, and the permeability of the intestinal barrier has also improved [187]. According to the European Crohn’s and Colitis Organisation, probiotics might be effective in inducing the remission in UC; however, they do not have better effects than mesalamine in terms of remission maintenance [188]. Probiotic treatment is also ineffective in CD [188]. Alterations in the serum levels of cytokines, such as an increase in IL-10 and a decrease of IL-6, without changes to TNF, in MS patients have been reported [189]. RA patients experienced a decrease in IL-6, IL-1β, and TNF and an increase in IL-10 [190]. Changes in cytokine levels might cause alleviation of pain and inflammation, and this could contribute to the improvement of sleep quality; however, more studies on the subject would be desirable.

Probiotic administration might also directly influence sleep quality and structure. VSL3, a probiotic drug, is able to elevate butyrate level, which increases time spent in NREM, while reducing the number of REM episodes in rats [191,192]. Similarly, prebiotics increasing *L. rhamnosus* administered to mice in their early life were shown to increase time spent in NREM (this effect was not long-lasting) [193]. Those prebiotics also increased REM sleep duration after a stressful situation [193].

Probiotic therapy might find application as adjuvant treatment in immune-mediated diseases. It is able to yield clinically significant benefits and has negligible side effects.

### 5.5. Anti-Inflammatory Treatment

Steroids, such as prednisolone, hydrocortisone, and prednisone, are commonly used drugs in inflammatory diseases, such as RA, IBD, psoriasis, and SLE. They are effective in attenuating inflammation; however, they might have a negative influence on sleep quality [97]. Symptoms of insomnia are commonly seen as a side effect. According to Ananthekrishan et al., the use of steroids is associated with sleep disruptions [97]. However, in a study performed by Sochal et al., the results of questionnaires assessing sleep quality, daytime sleepiness, and insomnia symptoms were not influenced by the steroid therapy [9]. Short-term use of dexamethasone was associated with changes to the sleep architecture, such as decreased sleep time, REM length, increased REM latency, and SWS length [194].

There is limited research on the influence of immunosuppressive immunomodulators, such as 6-mercaptopurine or azathioprine on sleep; however, current studies show that they do not influence sleep quality [57,195].

Anti-inflammatory treatment, which is often necessary in immune-mediated diseases, can modify the structure of sleep, whereas steroids appear to have a negative effect on sleep, monoclonal antibodies tend to improve its quality.

### 5.6. Continuous Positive Airway Pressure

CPAP is a well-established treatment for OSA. It is very effective in preventing the airways from collapsing, which prevents hypoxia and awakenings, thus restoring proper sleep architecture. As was already mentioned, it might reduce the risk of developing immune-mediated diseases, as well as lower the cortisol level. However, the influence of CPAP therapy, as well that of OSA itself on systemic inflammation, is controversial, as was presented by Unnikrishnan et al. in their review [196]. Based on recent findings, CPAP does not appear to reduce inflammatory markers [196]. Thus, an apparent decrease of risk for the development of certain immune-mediated diseases could potentially be attributed to the prevention of chronic hypoxia and associated consequences, but more studies on the subject are needed.

### 5.7. Summary

Therapy of patients with chronic inflammatory diseases requires addressing sleep problems, as they may affect the course of the underlying disease. Probiotics, melatonin, and synthetic agonists of MT1 and MT2 might be an interesting treatment option, due to negligible side effects; however, major clinical improvement should not be expected. Benzodiazepines, similarly to Z-drugs (zopiclone, eszopiclone, zaleplon and zolpidem), if possible, need to be avoided due to addictive potential or utilized only for short periods. In patients with concomitant depressive symptoms, antidepressants, such as trazodone, might be prescribed. Orexin receptor antagonists are a promising perspective but require further studies.

## 6. Conclusions

To summarize, there is a highly complex and bidirectional relation between sleep and immunity. Patients with immune disorders appear to be at a higher risk of conditions such as insomnia disorder or RLS, whereas sleep disorders, in turn, appear to affect the course of immune diseases, leading to an earlier relapse or onset. Since the prevalence of immune and sleep disorders seems to be on the rise, there is also a need to develop a better, more personalized treatment for those patients. For this reason, expanding the knowledge about the association between sleep immunity is vital from the clinical perspective and surely will aid in the therapy of patients with immune and chronic inflammatory diseases.
